# Her Heart's Mistake

**Published:** 1889-05-04

**Authors:** E. D. Cumming

**Affiliations:** Author of "An Ordeal by Silence"


### Her Heart's Mistake.

By E. D. Cumming, Author of "An Ordeal by Silence."

*(Continued from page 47.)*

### Chapter III.—*(Continued.)*

Fortune favoured him at dinner, for Captain Bairdsley was his neighbour, and seemed bent upon showing him all the attention he could. He talked well, but his conversation for the most part turned upon his own doings in various parts of Europe—where he had been, the exalted people he knew, the great houses at which he had been a visitor, and the high interest possessed by his friends. He did not speak boastingly of his titled acquaintances; he passed them lightly over, mentioning names casually, as though he wished it to be understood that the "upper ten thousand" was his native sphere. The Doctor listened, but, while he gave him no encouragement, he did not attempt to lead him into other channels. He was biding his time to discover whether he had any mental qualifications which would enlist his own real sympathy, and show him to be something more than a social butterfly. The conversation veered round to politics—a subject less dangerously contentious in India than at home—but beyond the questions bearing directly upon his own profession, Captain Bairdsley knew nothing, and admitted his lack of interest in national affairs. The class of literature he affected did not do much to recommend his tastes. Charles Lever, James Grant, Mounteney Jephson, and Mrs. Arkwright's favourite authoresses appeared to form his library. Excellent books in their way, but when the Doctor enquired whether he knew this work or that, which he had read himself, and knew to be books appreciated by men who think, he found that the Captain was out of his depth. He had begun such and such a volume, but found it too deep to be interesting. He had dipped into that author, but had not been able to see what a fellow could find to interest him in his writings. Dr. Harrison left the mess-house that night feeling that in two hours he had learned to know Captain Bairdsley as well as if he had known him for as many years. He was disappointed with the man. There was nothing in him apparently but what he had seen through his conversation at dinner. He was well-bred and gentlemanly, but had no depth of character. He had none of those rarer mental gifts which would have drawn the Doctor to him, and made the Doctor welcome him as a husband for Kate. He was good-looking, well behaved, and rich—nothing more, but perhaps, in this world of imperfections more could not be expected of one man. And as Kate's father lay down on his pillow that night he reflected that if she saw in him the qualifications which represented her ideal, he had seen nothing in him which could justify a parent in throwing obstacles in the way.

### Chapter IV.—Captain Bairdsley makes up his Mind.

A bareheaded native was running, as only a frightened native can run, across the compound of a certain small bungalow, pursued by an accurate and heavy fire of boots, which some strong-armed individual was delivering from the verandah.

"Get out, you scoundrel! Jao, you blackguard! Look at that *boot*" (boot hurled with great energy); "new three months ago, and ask me for your tullub again, if you dare! Tullub, indeed! *There's* your month's pay!" (another boot).

These, and a torrent of similar remarks couched in stronger language, greeted the ears of Captain Phelps one evening as he strolled over from the messhouse in time to see the flight of the aforesaid native.

"Bairdsley dismissing his bearer," said he to himself, putting his glass in his eye to watch the aërial course of a final missile; "I never knew such a fellow."

"I observe that your last new attendant is adding his name to the retired list," he said drily, coming into the bungalow where his friend stood, fuming with passion. "I fear that if you continue at the present pace you will soon exhaust the supply. Three bearers every five weeks will drain the labour market before long if you keep up your average."

"I don't know how anyone *can* put up with their impenetrable stupidity," replied Bairdsley, angrily; "I can't."

"What has he been doing?" enquired Captain Phelps, sinking into a chair and drawing out his cheroot case. "You appear to have hurt his feelings," he added, nodding at a group of servants on the road, in whose midst the boot-hunted bearer was gesticulating wildly, with his long unwound turban in his uplifted hand.

"Doing!" ejaculated Bairdsley. "Just look here, Phelps, I gave him three pairs of brown sand shoes to clean, and the idiot blacked them all. It's enough to rile a saint. I hope I've hurt something more than his feelings—confound him!"

"I suppose you told him *how* to clean them?" ventured his companion quietly.

"It's his business to know how to clean boots, I presume."

"Is it fair to expect a man to know how to treat things which, in all probability, he has never seen before, Bairdsley? If you won't take the trouble to learn a few words of the language, it's rather rough on your men to lick them for misunderstanding your orders."

"If they knew their work, orders wouldn't be necessary," retorted Bairdsley. And this being an unanswerable argument, Captain Phelps made no reply.

"I must get another of the creatures, I suppose," said the angry man, after a pause.

"You must get another—if you can," replied Captain Phelps.

"What will be the difficulty, pray?"

"The fact is simply this, my dear fellow. You treat your servants so badly that you will get yourself disliked, as someone puts it. Let's see: your first man you dismissed because he tore your mosquito curtain and you got bitten. That may have been right, but you ought not to have kicked him, and you ought to have paid him his wages. Then number two, whom you caught using your hairbrushes upon his own head, and slippered—I think I hear the smacks you gave him now. To your proceedings on that occasion I can't object. I should have thrashed him myself under the circumstances; but, again, you ought to have given him his pay. And to-day departeth the fleet-footed blacker of brown shoes with precipitate haste. I'd advise you to send your khit after him with his tullub if you don't want to be boycotted. You catch a native on his tenderest point when you withhold his pay."

"Do you mean to say that they won't come to me?" asked Bairdsley.

"No man worth his salt will, if you get a bad name among them."

"I'll have no more of them at all," said Bairdsley. "I'll take an English servant from my own company and be properly served."

"An English man-servant," said Phelps, thoughtfully, lying back in his chair, and gazing at the canvas ceiling. "I think I see him down at the dhoby tank getting master's clean clothes from the wash, and struggling among a crowd of bearers on the same errand. I see the patient British soldier lying on his back on the verandah, in a dog-sleep all day, ready to spring up at any moment and get master a whiskey-and-soda or a match. I see him squatting at the foot of master's camp-bed shampooing his feet by the hour to put him to sleep. I don't think Tommy Atkins would take kindly to the business, Bairdsley. Better try another nigger and treat him a little more reasonably."

"It's all very well to say treat them reasonably when you have good servants yourself! You forget that I can't speak the language, and that the fellows take advantage of it."

"Oh, no; I don't forget it, Bairdsley," answered Captain Phelps, with a serio-comic shake of the head; "you never give me a chance." This was strictly true; the quantity of opprobrious English which his friend daily lavished upon his domestics was very considerable.

"Shall I tell my bearer to bring you another man?" he enquired civilly.

"If you will," growled Bairdsley, feeling rather ashamed of himself; "I suppose I must have one."

"I'll make him order a dozen," answered Phelps gravely; "that will keep you going for a few months."

"Thank you; and when you have done talking bosh, we may as well go over to the club."

"I have quite finished," replied the other, who was one of those imperturbably good-humoured men whose temper nothing could ruffle. "I'll just put on another coat if you'll wait for a minute."

"I wonder if she has as much bother with her servants as I have with mine," speculated Captain Bairdsley to himself as he stood waiting for Captain Phelps to join him. "They seem to worship the ground she walks on, though she can't string three words of Hindustani together. She would be terrible woman in a rage, I suspect."

Kate Harrison might have been terrible when her ire was roused, but it required a great deal more to anger her than it did her lover. And her admiration for him would not have been increased had she seen his childish outburst of temper this afternoon.

Captain Phelps came out, and they walked amicably down to the club, where a large circle of men—civilians, soldiers, young army surgeons, and police officials—were discussing some proposal which had been made.

"The ground is much too hard just now," objected one man. "You might as well try to hunt at home after twenty degrees of frost."

"I don't see why it should be too hard for a few short races, when we play polo every day," remarked another.

"We needn't have steeplechases on the card either. It *is* too hard for jump-races, perhaps," said a third.

"What is the question before the House?" enquired Captain Phelps of Mr. Strachan, as he and Bairdsley joined the group.

"Colonel Tenterdale thinks the station is going to sleep for want of excitement, and has proposed that we get up a sky meeting," replied the Deputy-Collector.

"Why, certainly!" assented Captain Phelps. "The Colonel's quite right. We've had capital sky meetings in the hot weather often before now."

"What do you call a 'sky meeting,' Phelps?" asked Bairdsley.

"Races got up at a few day's notice. I believe the term originated in some part of India where the rainy season lasts for several months. When the sky cleared for a day or two they seized the opportunity for a little racing; hence the name. Now, any informal day's sport for which the animals receive no particular training is called a 'sky' meeting."

"It would be great fun," said Bairdsley, who was as good a sportsman and rider as need be. "I shall certainly go for it if it's put to a vote."

"I don't imagine there will be many objectors. No one need enter his horses or ponies if he doesn't want to."

"I shall enter my pony," said Mr. Wentworth, unintentionally taking advantage of a momentary silence to make the announcement in a very decided tone of voice.

"Hear, hear," shouted one of his brother officers. "Mr. Tops' well-known country-bred pony 'Hair-trunk—'"

"We will go inside and see if we can't draw up a little programme," said Colonel Tenterdale, interposing, much to Mr. Wentworth's relief. "Who were on our last committee?"

Three or four men edged forward and signified that they had had that distinction at the Meerut Christmas Sky Races, and intimated their willingness to assist now in organising an afternoon's sport.

"Will you join us, Bairdsley?" asked the Colonel. "I believe you have had some experience in these matters at home."

Captain Bairdsley accepted the invitation, and the committee thus completed, adjourned to the reading-room of the club."

"We must send round the hat as usual, I suppose," sighed Mr. Strachan, as the half-dozen men seated themselves at a table. "Khitmugar! Bring a sheet of paper and a pencil."

"We had better set the example by putting down our own mites first," remarked Colonel Tenterdale, as the servant brought the writing materials. "Shall we limit the subscriptions?"

"Hardly necessary, I think," said Major Arkwright, a small wiry man, sunburnt almost to the complexion of a native. "We don't want much money, and fellows aren't likely to be extravagant over meetings of this kind."

"Very true," assented the Colonel. "If we put down a gold mohur apiece, it will be quite enough, and others can give five rupees or what they like."

The others agreed, and having written their names on the list as subscribing sixteen rupees—the nominal equivalent of the rarely seen "gold mohur"—each, the business of drawing up what Captain Phelps called the "menu" was proceeded with.

It was a very simple affair; a half-mile race "for all horses which had never won a stake of two hundred rupees or over, weight for age and class"; and three others of various lengths for horses and ponies were decided upon without difficulty. But then there was a pause; four races would not fill up the afternoon, or rather the evening, and something more was necessary.

"We ought to have a jump-race," said Mr. Strachan, but I'm afraid that men won't enter their horses when the course is so hard as it is now."

"May I suggest that if the ground on the landing side of fences were well hoed up, the chief objection would be removed?" put in Captain Bairdsley.

"We might do that," assented the Colonel, "but before we consider what sort of race we shall have, we must estimate the amount of money we shall be able to collect for the prize fund and expenses."

"I'm afraid last year's sky meetings won't give us a very just idea of the probable sum," said Mr. Strachan; "there are a lot of fellows away from the station just now. All the moneyed men who can get away are off to Cashmere or the hills, and only the paupers are left," he added, with grim humour.

Captain Bairdsley, who was sitting next to his commanding officer, whispered something in his ear, to which the latter first politely objected, but after a little pressure gave way.

"Captain Bairdsley has most kindly offered to increase his contribution, so that we need not let monetary considerations deter us from producing such a card as we should like," said the Colonel, addressing the committee. "We won't take advantage of his generosity until we have sent round the list, but I think, on the strength of it, we may, at all events, have a jump-race."

Opinion was divided as to what it should be, and eventually a hurdle race was added. Bairdsley expressed himself strongly in favour of that, in preference to the steeplechase proposed by some of the men, and in deference to his liberality the opposing faction gracefully gave way.

He had his own reasons for advocating "hurdles." Miss Harrison had frequently given him glowing accounts of the speed of her Arab mare "Baroness" and its leaping powers; and he, whose experienced eye had taken in at a glance the indications of speed and strength in the animal's shape, had praised its promising appearance, and recommended her to try what it could do on the turf. The mare had never started in a race, but Kate had been fired with ambition to have more than an onlooker's interest at the Meerut racecourse, and Bairdsley knew that this opportunity of testing her favourite's capabilities would be welcome to her. The Arab was hardly fast enough to run with a chance of success on the flat, and had had no education in negotiating the various obstacles which adorn a steeplechase course. But its cleverness "over sticks" pointed to the sphere in which it was most likely to win laurels, and the Captain, using his purse to give weight to his vote, made a point of having a race of the required kind placed on the programme. He had ridden and won his share of races at home, and he fondly nursed the thought that Kate would take double pleasure in "Baroness'" performance if he wore her colours and steered the mare himself.

"I'll go over to the ladies' club and tell her what we have arranged," he said to himself, as the committee meeting concluded its task.

He strolled over to the drawing-room, but on reaching the verandah was disappointed to learn from her aunt that Kate had gone home complaining of headache. He remained for half an hour chatting with Mrs. Brent, with whom, as Miss Harrison's aunt, he was anxious to stand well, and then having received an invitation to breakfast at the corner bungalow on the following morning, bent his way towards his own quarters.

Captain Phelps' servant came to his room to present a new bearer, whom he had that evening brought from the bazaar, and having perused the man's characters, and discovered that he could speak a little English, Bairdsley lay down in his chair on the verandah for a smoke.

This evening he lay in his chair, wondering at the strange chance which had found him in this remote spot what he had never seen in all his wide circle of acquaintance in England—the woman he could love, the one woman whom he wished to call his wife. Perhaps, the unaccustomed life of solitude, the long hours of idle dreaming thought had led him to yearn for a wife's companionship. George Bairdsley was impulsive, and his present existence was well nigh insupportable to him. He had conceived in five short weeks a passion which sometimes surprised his calmer self; and to-night, when he left his chair to go and get ready for mess, he did so with the fixed resolve to lay his hand and fortunes at Kate Harrison's feet the next day.

The forthcoming races were the all absorbing topic of conversation at dinner that night, but he had lost interest in them though before he had finished his soup he had been asked and had promised to take mounts in every flat race on the ensuing Thursday, for which day they had been fixed. His silence and absent-mindedness were remarked, and more than one observant man rightly guessed the cause. He was taking council with himself upon the words in which he should make his declaration to Kate, and forcasting the answer he should receive.

*(To be continued.)*

